# Novel analysis of the Harderian gland transcriptome response to Newcastle disease virus in two inbred chicken lines

**DOI:** 10.1038/s41598-018-24830-0

**Published:** 2018-04-26

**Authors:** Melissa S. Deist, Rodrigo A. Gallardo, David A. Bunn, Terra R. Kelly, Jack C. M. Dekkers, Huaijun Zhou, Susan J. Lamont

**Affiliations:** 1 Department of Animal Science, Iowa State University, Ames, Iowa USA; 2 Department of Population Health and Reproduction, School of Veterinary Medicine, University of California, Davis, California, USA; 3 Department of Animal Science, University of California, Davis, California, USA; 4 One Health Institute, University of California, Davis, California, USA

## Abstract

Behind each eye of the chicken resides a unique lymph tissue, the Harderian gland, for which RNA sequencing (RNA-seq) analysis is novel. We characterized the response of this tissue to Newcastle disease virus (NDV) in two inbred lines with different susceptibility to NDV across three time points. Three-week-old relatively resistant (Fayoumi) and relatively susceptible (Leghorn) birds were inoculated with a high-titered (10^7^EID_50_) La Sota strain of NDV via an oculonasal route. At 2, 6, and 10 days post infection (dpi) Harderian glands were collected and analyzed via RNA-seq. The Fayoumi had significantly more detectable viral transcripts in the Harderian gland at 2 dpi than the Leghorn, but cleared the virus by 6 dpi. At all three time points, few genes were declared differentially expressed (DE) between the challenged and nonchallenged birds, except for the Leghorns at 6 dpi, and these DE genes were predicted to activate an adaptive immune response. Relative to the Leghorn, the Fayoumi was predicted to activate more immune pathways in both challenged and nonchallenged birds suggesting a more elevated immune system in the Fayoumis under homeostatic conditions. Overall, this study helped characterize the function of this important tissue and its response to NDV.

## Introduction

The Harderian gland is a small tissue located behind the eyes of the chicken. In a previous study of adult chickens, the average weight was found to be 84.4 mg^1^. The major functions of this tissue have not been fully characterized but are thought to involve lubrication of the nictitating membrane^2^ and the local immune response, serving as a site of antibody production^3^. The cells of the Harderian gland are both bursa- and thymus-dependent^4,5^. Large numbers of heterophils and plasma cells accumulate in the chicken Harderian gland by two weeks of age^2^, and this is a location of terminal B cell maturation^6^. Antigenic stimulation of the Harderian gland also increases the number of plasma cells^7,8^. Although some studies have shown evidence of low numbers of T cells residing in the Harderian gland^9,10^, others saw an increase in T cell numbers after infection with Newcastle disease virus (NDV)^11,12^. There have been several studies on this unique tissue in the past 50 years, but there has been very little recent characterization of the Harderian gland with new technologies. To the authors’ knowledge, the Harderian gland transcriptome has never been reported prior to this study.

NDV is a major global problem in poultry. This virus can cause high levels of mortality when birds are not vaccinated, or vaccinated improperly^13^. Eye-drop vaccination is a common method and it is unknown how the transcriptome of the closest immune tissue responds to vaccination.

Genetics plays a role in the chicken’s response to NDV^14–16^ and to the live virus vaccine^17–19^. A previous study has identified two inbred chicken lines that differ in their susceptibility to NDV^17^. The Fayoumi (relatively resistant) had significantly lower viral load in the lachrymal fluid at 6 dpi, numerically higher serum antibodies at 10 dpi, and significantly lower viral transcript counts in the tracheal epithelial cells than the Leghorn (relatively susceptible) after challenge with lentogenic NDV^17^. Studying these lines’ response to NDV in the Harderian gland can offer insights into both vaccine development and host resistance. We predict these two lines will respond differently to NDV in the Harderian gland transcriptome in terms of viral transcript counts, numbers of differentially expressed genes (DEG), and activated pathways. These differences will help to identify possible mechanisms of NDV resistance in the chicken.

## Results

### Summary statistics of the RNA-seq reads

Overall, no bias was shown due to sequencing or mapping differences among groups (Table 1). Each sample was composed of two technical replicates, for which the raw counts for each transcript were summed. The average transcriptome coverage percentage, i.e. the percentage of transcripts with at least 1 count, was 37.0%, nearly 2% lower than that of the lung^18^. The percentage of reads that mapped to the reference genome on average across all samples was 91.17% (Table 1).

**Table 1:** Summary Statistics of RNA-seq reads.

	Raw Reads	Filtered Reads	Mapping %
Average	26,110,910	21,274,532	91.17
Leghorn	23,376,646	18,941,376	91.42
Fayoumi	28,964,055	23,709,129	90.91
Challenged	26,960,703	21,908,635	91.13
Nonchallenged	25,224,170	20,612,859	91.21

### Viral sequences were detected in the unmapped reads of challenged birds

**Figure 1:**
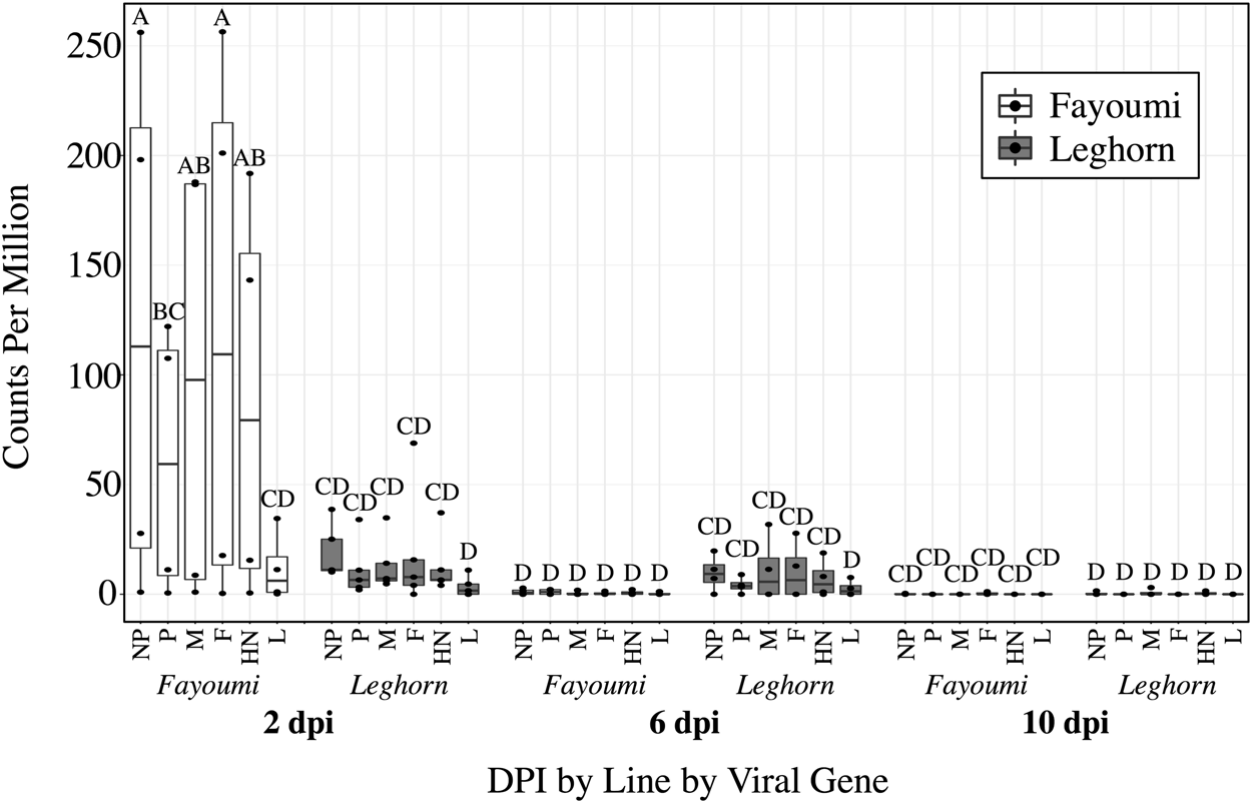
Viral transcripts detected in the unmapped reads of the challenged Fayoumis and Leghorns at 2, 6, and 10 days post infection. Points within each box plot represent the counts per million (cpm) (y-axis) of each viral gene from 3′ to 5′: Nucleoprotein (NP), Phosphoprotein (P), Matrix protein (M), Fusion protein (F), Hemagglutinin-neuraminidase (HN), or Polymerase (L), in the challenged Fayoumi (white) or Leghorn (grey), at 2, 6, or 10 dpi (x-axis). A Student’s t-test connecting letters report was generated. Box plots that do not share letters are significantly different from each other. For each group n = 4, except the Leghorns at 2 dpi (n = 5) and the Fayoumis at 10 dpi (n = 3).

The main effects of line (p < 0.0001), dpi (p < 0.0001), and viral gene (p = 0.015) all had a significant impact on the viral counts per million reads (cpm) (Fig. 1). There was also a significant interaction between line and dpi (p < 0.0001). The Fayoumis at 2 dpi had significantly higher viral cpm than the Fayoumis or Leghorns at any time point, however, at 6 dpi, only the Leghorns had detectable virus (Fig. 1). By 10 dpi, both breeds showed no detectable virus (Fig. 1).

### The principal component analysis plot shows clear clustering by line

**Figure 2:**
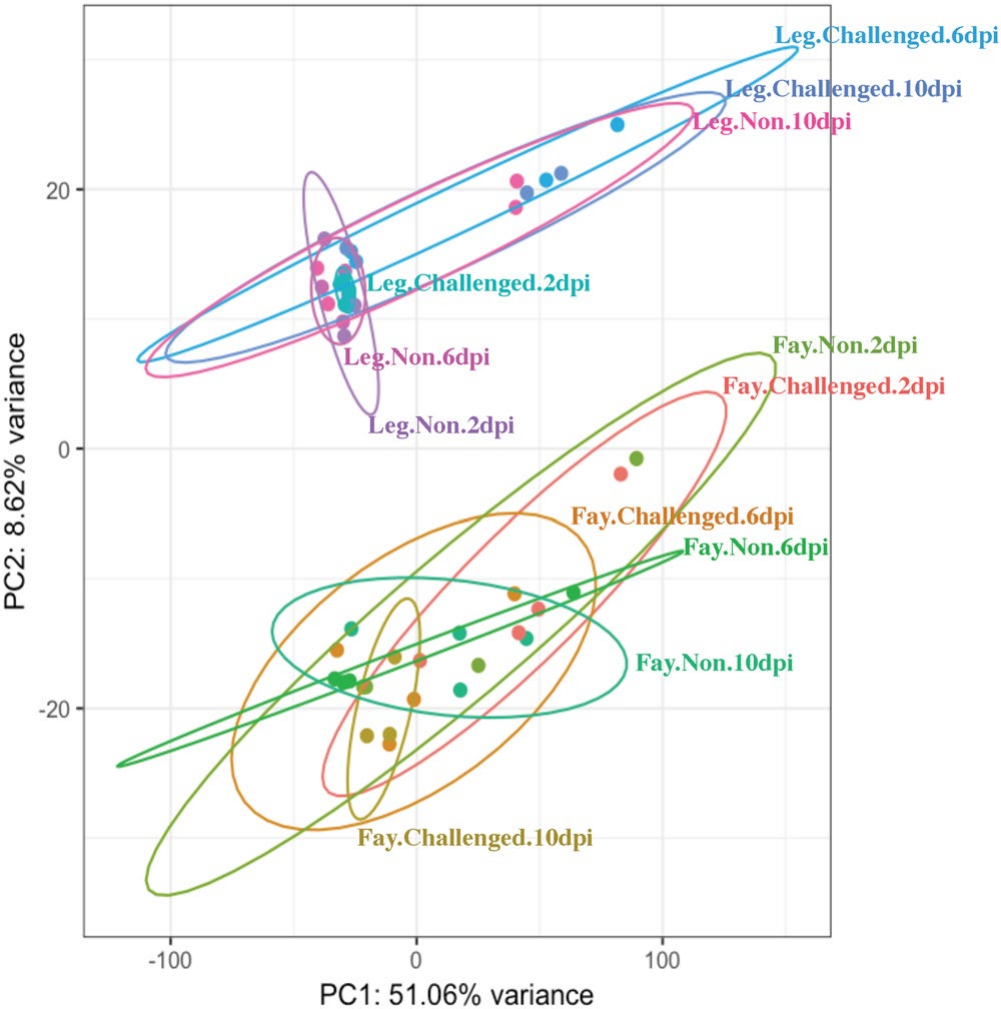
Principal component analysis plot generated by pcaExplorer. Each dot represents a sample colored to separate by line (Fayoumi: Fay; Leghorn: Leg), challenge status (Challenged; Nonchallenged: NON), and time (2, 6, and 10 dpi). Ellipses were drawn around each treatment group with 95% confidence. The top 5000 genes were used to calculate the principal components. Principal component 1 (PC1) accounts for 51.06% of the variance in the Harderian gland transcriptome. PC2 accounts for 8.62% of the variance and clusters clearly separate by line.

The principal component analysis (PCA) plot generated by pcaExplorer shows that principal component (PC) 2 separated the samples clearly by line and accounted for 8.6% of the variance seen in the Harderian gland transcriptome (Fig. 2). PC1 accounted for a large portion of the variance (51.1%), however, samples did not clearly cluster based on any known parameters. PC1 may be related to dpi because some time points clustered very closely on PC1 (Fig. 2). The differentially expressed genes between the nonchallenged birds over time within each line are included in Supplementary Table S1.

### Challenged vs. nonchallenged birds by line and time

**Figure 3:**
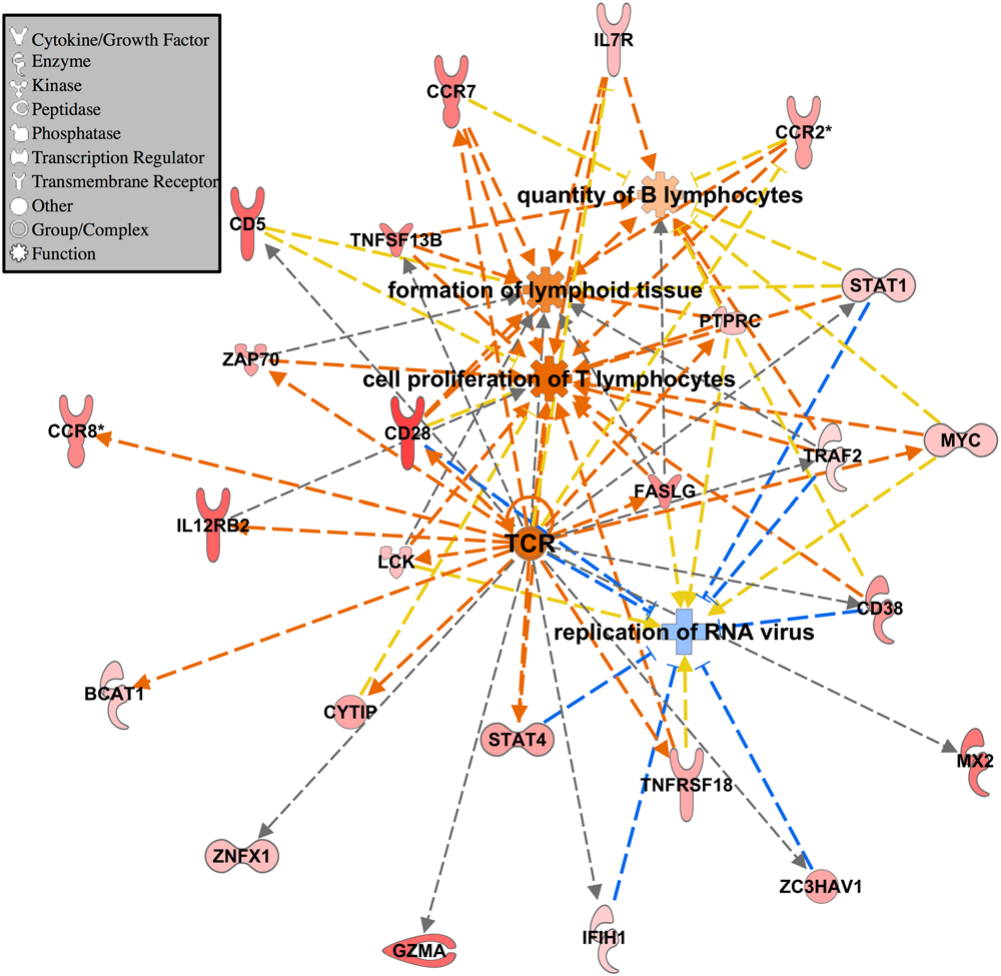
IPA analysis for the challenged vs. nonchallenged DEG in the Leghorn at 6 dpi. Data were analyzed through the use of IPA (QIAGEN Inc., https://www.qiagenbioinformatics.com/products/ingenuity-pathway-analysis). All genes input into IPA had FDR <0.05 and absolute LFC >1. The larger the positive LFC for each gene, the darker the red fill. Genes and functions are connected with dashed lines that represent predicted activation (orange), an unknown relationship (grey), or a relationship that does not agree with the database (yellow). Orange fill represents a predicted activation, and blue, predicted inhibition. Genes in figure were grouped by the upstream regulator TCR, diseases and functions were added using the grow function in IPA (p < 0.0001). Gene shapes indicate the molecule type (see legend).

The challenge resulted in few DEG at most time points within each line (Table 2). Of the few DEG, several have known immune function including STAT1, Mx1, and TLR3, which were all upregulated due to challenge (Table 2). The entire list of DEG between the challenged and nonchallenged birds within each line and time are included in Supplementary Table S2. The Leghorns at 6 dpi had much larger numbers of DEG (Table 2), which may be related to lack of viral clearance at 6 dpi in the Leghorns as shown by higher viral cpm values (Fig. 1). Pathway analysis was performed to further characterize the response to challenge in the Leghorns at 6 dpi because of the large number of DEG (Table 2). The top five canonical pathways with a z-score >0.01 generated by IPA included the Th2 Pathway, Th1 Pathway, iCOS-iCOSL Signaling in T Helper Cells, CD28 Signaling in T Helper Cells, and PKCθ Signaling in T lymphocytes. All of these pathways were predicted to be activated and involved the T cell response. The B Cell Development Pathway was also significant and predicted to be activated, but was not among the top 5 canonical pathways. The upstream regulator T cell receptor (TCR) was predicted to be activated due to the increased expression of the genes (Fig. 3). The expression levels of these genes were also predicted to activate quantity of B lymphocytes, formation of lymphoid tissue, and cell proliferation of T lymphocytes and inhibition of replication of RNA virus (Fig. 3). Thus, in response to NDV infection, at 6 dpi, the Leghorns were activating both the cell mediated and humoral arms of their adaptive immune response in this unique tissue.

**Table 2:** Challenged vs. nonchallenged down and upregulated genes within each line and day.

Line	DPI^a^	Downregulated^b^	Upregulated^c^	Immune Related Genes
Fayoumi	2	1	9	TNFRSF6B^c^, IFI6^c^, ZNFX1^c^, Mx1^c^
Leghorn	2	2	17	C1S^b^, MPEG1^c^, TLR3^c^, IFI6^c^, ZNFX1^c^, Mx1^c^
Fayoumi	6	0	0	‒‒
Leghorn	6	57	623	C1S^c^, ICOS^c^, STAT1^c^, CCR7^c^, LITAF^c^, ITK^c^
Fayoumi	10	5	18	IGFBP2^c^, DNAJB11^b^
Leghorn	10	0	0	‒‒

^a^Days Post Infection; ^b^downregulated due to challenge FDR <0.05; ^c^upregulated due to challenge FDR <0.05; TNF Receptor Superfamily Member 6b (TNFRSF6B); Interferon Alpha Inducible Protein 6 (IFI6); Zinc Finger NFX1-Type Containing 1 (ZNFX1); MX Dynamin Like GTPase 1 (Mx1); Complement C1s (C1S); Macrophage Expressed 1 (MPEG1); Toll-like receptor 3 (TLR3); Inducible T-Cell Costimulator (ICOS); Signal transducer and activator of transcription 1 (STAT1); C-C chemokine receptor type 7 (CCR7); Lipopolysaccharide Induced TNF Factor (LITAF); IL2 Inducible T-Cell Kinase (ITK); Insulin Like Growth Factor Binding Protein 2 (IGFBP2); DnaJ Heat Shock Protein Family (Hsp40) Member B11 (DNAJB11).

### Genetic line differences were apparent in the Harderian gland transcriptome in both challenged and nonchallenged birds at all times

**Figure 4:**
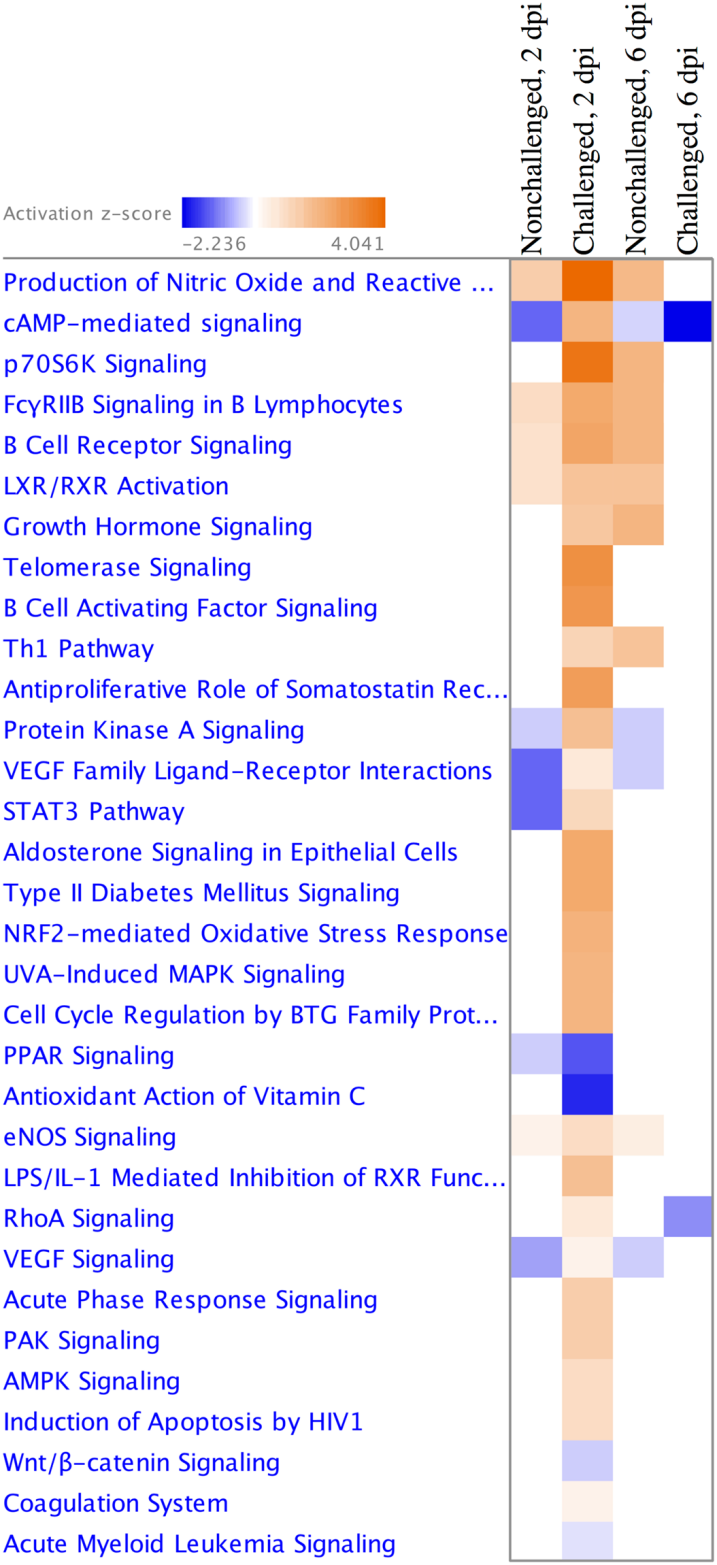
Pathways more differentially activated between the lines within each treatment group at 2 and 6 days post infection. The Fayoumis and Leghorns were directly compared within each dpi and challenge group. The resulting differentially expressed genes with FDR <0.10 were used as input into IPA for pathway analysis. Contrasts from 10 dpi were removed because no pathways were significant. Heat map shows pathways predicted to be relatively more activated in the Fayoumi (orange) and Leghorn (blue). The more intense a square in the heat map, the higher the z-score and prediction confidence. White squares represent contrasts with no or too few DEG within that pathway to make a prediction (z-score = 0).

The entire list of DEG between the Fayoumis and Leghorns within each challenge group and time are included in Supplementary Table S3. The number of DEG at 2 dpi between the challenged Fayoumis and Leghorns was larger than between the age-matched, nonchallenged Fayoumis and Leghorns (Table 3). This suggested that their transcriptomes became more different after challenge with the virus, even though within-line contrasts of challenged vs. nonchallenged birds showed few DEG at this time (Table 2). The extremely tight clustering of transcriptomes of the challenged Leghorns at 2 dpi (Fig. 2) may have increased the ability to call genes as differentially expressed between the challenged Fayoumis and Leghorns. Overall, 58 genes were consistently differentially expressed in the Fayoumis and Leghorns at all times and in both challenged and nonchallenged birds. These DEG may be relevant to breed characterization. Somatotropin, for example, was always more highly expressed in the Fayoumis (Table 3) and has previously been associated with viral resistance to Marek’s disease in chickens^20^. Further analysis of the DEG between the Fayoumi and Leghorn revealed which pathways were more highly activated within each line (Fig. 4). Many inflammatory pathways were more activated in the challenged Fayoumis at 2 dpi, and this may be related to the amount of viral transcript counts present at that time. Interestingly, most of the pathways that were differentially activated between the two lines appear to be immune related (Fig. 4). Pathways in which the predicted activation was of different direction in challenged and nonchallenged birds are of particular interest.

**Table 3:** Fayoumi vs. Leghorn differentially expressed genes within each challenge group and time point.

Challenge Status	DPI^a^	More Highly Expressed In		Immune Related Genes
		Leghorn^b^	Fayoumi^c^	
Nonchallenged	2	181	196	TRAP1^b^, Somatotropin^c^, MZB1^c^, IFNG^c^
Nonchallenged	6	144	263	CFAP221^b^, Somatotropin^c^, JCHAIN^c^, CCR10^c^
Nonchallenged	10	87	87	Somatotropin^c^, DPT^c^
Challenged	2	817	1481	TRAP1^b^, MZB1^c^, JCHAIN^c^, CCR10^c^
Challenged	6	163	201	NFKBIZ^b^, IL18^b^, ZNF830^c^
Challenged	10	88	194	NFKBIZ^b^, TRAP1^b^, ACKR^c^, TNFAIP6^c^

^a^Days Post Infection; ^b^more highly expressed in the Leghorns FDR <0.05; ^c^more highly expressed in the Fayoumis FDR <0.05; TNF receptor associated protein 1 (TRAP1); marginal zone B and B1 cell specific protein (MZB1); interferon gamma (IFNG); cilia and flagella associated protein 221 (CFAP221); joining chain of multimeric IgA and IgM (JCHAIN); C-C motif chemokine receptor 10 (CCR10); dermatopontin (DPT); NFKB inhibitor zeta (NFKBIZ); interleukin 18 (IL18); zinc finger protein 830 (ZNF830); atypical chemokine receptor 2 (ACKR); TNF alpha induced protein 6 (TNFAIP6).

### Genes impacted by the challenge*line interaction are important

Genes that were significantly impacted by the challenge*line interaction may offer insights into mechanisms of disease resistance or vaccine readiness. At 2 dpi, genes significantly impacted by the interaction included: Down Syndrome Cell Adhesion Molecule (DSCAM), Glutamate Ionotropic Receptor NMDA Type Subunit 1 (GRIN1), Synaptosomal-associated protein 25 (SNAP25), and CaM Kinase Like Vesicle Associated (CAMKV). At 6 dpi, no genes were significantly impacted by the challenge*line interaction. Phenylalanyl-TRNA Synthetase Alpha Subunit (FARSA), Carboxypeptidase A6 (CPA6), Ribosomal Protein Lateral Stalk Subunit P2 (RPLP2), and ENSGALT00000073955 were identified as significantly impacted by the challenge*line interaction at 10 dpi.

### RNA-seq results were validated by high throughput qPCR

**Figure 5:**
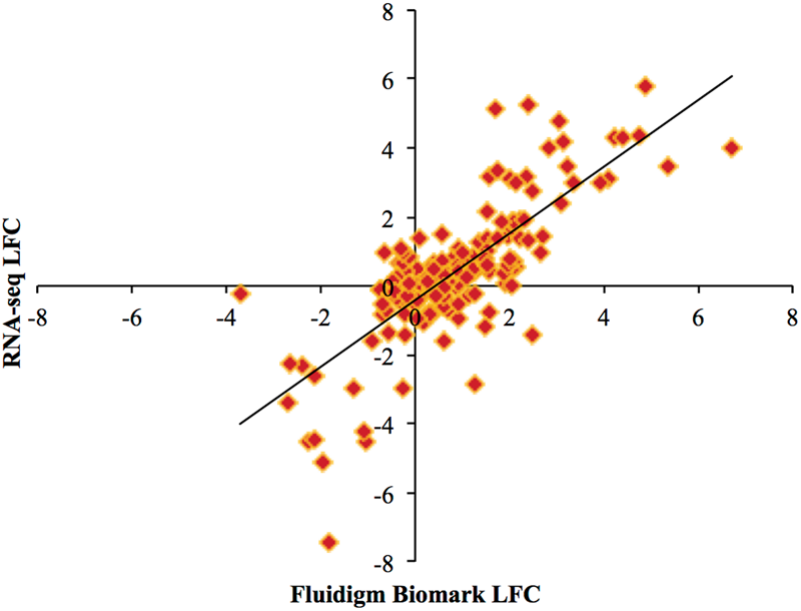
Scatterplot correlation between LFC estimated by RNA-seq and Fluidigm Biomark. Each point represents a gene’s log fold change (LFC) as measured by RNA-seq (y-axis) and Fluidigm Biomark (x-axis). Contrasts between challenged and nonchallenged birds within each line and within each time were used to generate this plot. A total of 32 genes were analyzed across these six different contrasts resulting in 192 data points. The correlation between the two technologies was r = 0.77.

Fluidigm Biomark was used as a method of high throughput qPCR to validate the RNA-seq methods by correlating the log fold change (LFC) calculated by both techniques. Primers were previously published^17^. The six challenged vs. nonchallenged contrasts (within each time and line), were used to compare the RNA-seq LFC with the Biomark LFC calculated via the −2^−ΔΔCT^ method (Fig. 5). The correlation (r = 0.77) was strong.

## Discussion

There was a large number of DEG between the challenged Fayoumis and Leghorns at 2 dpi. This was surprising, because there were few DEG in the contrasts of challenged and nonchallenged birds within-line at 2 dpi. The PCA can explain some of this result. The transcriptome of challenged Leghorns at 2 dpi clustered very tightly together with the two largest principal components (which together account for 60% of the variance), much tighter than the nonchallenged Leghorns at 2 dpi. The challenged Fayoumis clustered more tightly than the nonchallenged Fayoumis as well at 2 dpi. A multi-dimensional-scaling plot showed the same clustering patterns as the PCA plot (data not shown). Less within-group variation allows for the identification of more DEG. The PCA plot also showed the tight clustering of the nonchallenged Leghorns at 6 dpi, which may have allowed for the detection of more DEG when contrasting the challenged and nonchallenged birds at this time. The samples within Leghorn treatment groups tended to diverge with time, whereas in the Fayoumis, the samples clustered more tightly as time progressed across PC1. It is unclear what drives some treatment groups to cluster more tightly than others; PC1 accounts for a large portion of variance and cannot be identified from the study parameters.

Previous studies have shown the influx of plasma cells and T cells to the Harderian gland after infection with NDV^5,11,12^. In the Harderian gland, plasma cells have been shown to synthesize mainly IgA^5^, which has been detected as early as 4 dpi in the saliva of Leghorns^21^ after infection with NDV. However, no IgA was detected in the lachrymal fluid of the challenged Fayoumis or Leghorns at 2 & 6 dpi (data not shown).

The large number of DEG seen between the challenged and nonchallenged Leghorns at 6 dpi shows the activation of the adaptive immune response in these birds. These DEG predicted the activation of several T cell-related pathways. The activation of T cell-related pathways after infection was also seen in the trachea epithelial cells of these birds^17^, but not in the lung, which had no detectable viral transcript counts^18^. T cell-related pathways appear to be activated at sites of viral replication, which is likely a result of an increased T cell population. In another study, after infection with Hitchner B1 NDV strain, all T cell subsets increased at least 2-fold in the Harderian gland, and CD8+ T cells increased 6-fold^12^.

There might be a relationship between the large number of DEG and the viral transcript counts in the Harderian gland of the Leghorns at 6 dpi. Viral persistence in the Leghorns could cause the large DEG response at 6 dpi in the Leghorns that was absent in the Fayoumis, because the Fayoumis cleared the virus by 6 dpi. It is unknown, however, if the viral transcript counts correlate with the number of infectious viral particles. Surprisingly, the Fayoumis had significantly more detectable viral transcript counts, as estimated from the RNA-seq, at 2 dpi than the susceptible Leghorns in the Harderian gland. This result should be explored further to determine whether greater viral transcript counts is advantageous for mounting an appropriate immune response in the Harderian gland. Overall, the cpm for each viral gene detected in the Harderian gland was lower than that detected in the trachea epithelial cells of these birds^17^. The different cell composition of these tissues may be one of the causes. Likely, a higher percentage of cells were susceptible to lentogenic NDV replication in the trachea epithelial cells than the Harderian gland, as lentogenic NDV requires a protease found in epithelial cells to cleave the Fusion protein^22^. Also, unlike the trachea, in the Harderian gland the viral gene cpm did not decrease from 3′ to 5′ as would be expected^17^. The high cpm of the F and HN genes in the Harderian gland of challenged Fayoumi at 2 dpi may have facilitated the production of more neutralizing antibodies to these external structural proteins.

The two genetic lines clearly responded differently to the virus in the number of DEG and in the amount of detectable viral transcript counts. When the lines were directly compared, several immune related pathways differed in their relative activation. Relative to the Leghorns, the Fayoumis had more immune-related pathways that were in an activated state under resting conditions (nonchallenged), including eNOS signaling, Th1 pathway, LXR/RXR Activation, B cell receptor signaling, FcγRIIB signaling in B lymphocytes, and production of nitric oxide and reactive oxygen species. These pathways represent each facet of the immune system: innate, cell mediated, and humoral immunity. The relative activation of these immune pathways in the nonchallenged Fayoumis may be a reason for the low numbers of DEG found between the challenged and nonchallenged Fayoumis at the time points measured. Differences in immune related pathways of nonchallenged Fayoumis and Leghorns was also observed in the spleen^19^. It is unknown whether the reason for the Fayoumi’s resistant or Leghorn’s susceptible phenotype is due to their response to pathogen or their constitutive expression levels. The increased relative activation of immune related pathways under homeostatic conditions may be partially responsible for the favorable phenotype observed in the Fayoumis. The Fayoumis may be more equipped to handle a viral infection under resting conditions and, therefore, do not need to mount a large immune response to clear the virus, as assessed by changes in gene transcription.

The genes that were significantly impacted by the challenge*line interaction are of particular interest. Of those genes, some, including RPLP2, DSCAM, and CPA6, have been shown to have immune function. Flaviviruses require RPLP2 for infection, because it is necessary for viral translation^23^. In arthropods, DSCAM plays a crucial role in the innate immunity’s specificity^24^. In initial cancer stages, CPA6 mRNA is more highly expressed than in late stages^25^. These genes are of importance to those looking for mechanisms of disease resistance or the genetic impact on vaccination efficacy.

## Conclusions

To the authors’ knowledge, this is the first time the Harderian gland transcriptome has been analyzed using RNA-seq. The Harderian gland of two genetic lines showed very different responses to NDV in number of DEG and amount of detectable NDV. The pathways activated in the nonchallenged Fayoumi suggest a more elevated immune system relative to the Leghorn under resting conditions. The challenged Fayoumi had significantly higher viral transcript counts in the Harderian gland at 2 dpi but no longer had detectable viral transcript counts at 6 dpi, unlike the Leghorn. This suggested the Fayoumis might clear the virus more quickly than the Leghorns. The Harderian gland is a first responder tissue after eye-drop vaccination or aerosol transmission of the virus. This study established a foundation for future research to investigate this tissue’s unique role in host defense.

## Methods

### Animal experiment

This study was approved by the Iowa State University Institutional Animal Care and Use Committee (IACUC log number 1-13-7490-G) and the experimental design and methodology has been described previously^17,18^. All experiments were performed in accordance with the committee’s relevant guidelines and regulations. Two inbred lines, the Fayoumi (M 15.2) and Leghorn (GHs 6), from the Iowa State University Poultry Farm (Ames, IA), were used as a discovery platform to model relative resistance and susceptibility to NDV. Fayoumi and Leghorn breeders are a National Poultry Improvement Plan (NPIP) certified flock, tested and confirmed *Salmonella* and avian influenza virus free. Breeders of both lines are housed in the same building at the Iowa State University Poultry Farm (Ames, IA). At hatch, birds were placed in a biosafety level 2 facility. Chickens of both genetic lines were co-mingled throughout the study. Three weeks later, the challenged birds (n = 49) were inoculated with 200 μL of 10^7^EID_50_ of La Sota NDV via the ocular/nasal route, and the nonchallenged birds (n = 40) were inoculated with PBS in the same manner. All challenged and nonchallenged birds were negative for NDV specific antibodies prior to challenge. Challenged and nonchallenged birds were kept in separate rooms from hatch until the end of the experiment. Approximately one third of the chickens were euthanized at 2, 6, and 10 dpi for tissue collection, the Harderian gland was removed, and placed into RNAlater (ThermoFisher Scientific, Waltham, MA). In the same week, tissues were processed and then stored at −80 °C. Each treatment group included four biological replicates for transcription analysis with the exception of the Leghorns at 2 dpi (5 challenged, 3 nonchallenged) and 3 challenged Fayoumis at 10 dpi. Total RNA was isolated from these samples using the RNAqueous kit (Thermo Fisher Scientific, Waltham, MA) and was DNase treated using a DNA-free kit (Thermo Fisher Scientific, Waltham, MA). All samples had an RNA quality number >8.0, as measured using the *PROSize*® Data Analysis Software and the Fragment Analyzer™ Automated CE System (Advanced Analytical Technologies, Inc., Ankeny, IA). The isolated RNA from each sample was used to independently construct two cDNA libraries for each biological replicate using the high-throughput protocol in the TruSeq RNA sample preparation guide (v2; Illumina, San Diego, CA). Libraries were validated using the Fragment Analyzer™ Automated CE System (Advanced Analytical Technologies, Inc., Ankeny, IA) and 100 bp single end reads were sequenced on the HiSeq2500 (Illumina, San Diego, CA) at Iowa State University DNA Facility (Ames, IA). The sequencing data are available in the ArrayExpress database at EMBL-EBI (https://www.ebi.ac.uk/arrayexpress) under accession number E-MTAB-6038.

### RNA-seq pipeline

The sequence data were taken through a standard pipeline for analysis using FASTX, TopHat2^26^, and HTSeq^27^ as described previously^18^. A chi-square test was performed on the HTSeq output for each pair of technical replicates to confirm the technical replicates had minimal differences. Then the raw counts were combined for each pair of technical replicates. One technical replicate was discarded because only 10% of its reads mapped to the chicken reference genome, indicating technical failure.

Reads that did not map to the chicken reference genome were analyzed further as described previously^17^. These unmapped reads from each technical replicate were mapped to the La Sota genome (GenBank accession number JF950510.1) using BWA^28^ and the mapped reads were counted with HTSeq^27^. The technical replicates’ raw counts for each viral gene were summed for each biological replicate. The counts per million for each viral gene for each biological replicate were statistically analyzed in JMP statistical software (JMP Group Inc., San Francisco, CA) using a standard least squares, effect leverage full factorial test that accounted for line, dpi, gene, and their interactions.

### Principal component analysis

For data visualization, pcaExplorer^29^ was utilized. The dds function from DESeq2^30^ was used for normalization, accounting for line, challenged, dpi, and sex in the model, and the rlogtransformation in pcaExplorer was used. The 5000 transcripts that contributed the most variance were used to calculate the variance associated with the principal components.

### Differential expression analysis

For differential expression analysis of the count data, the generalized linear model in edgeR^31^ accounted for line, challenge, and dpi to determine the number of DEG (False Discovery Rate (FDR) <0.05). Contrasts were written to compare challenged and nonchallenged birds within each line and time, Fayoumis and Leghorns within each challenge group and time, and the challenge*line interaction at each time.

The edgeR output, including LFC and FDR for each transcript for each contrast, was input into Ingenuity Pathway Analysis (IPA; QIAGEN Inc., Redwood City, CA, https://www.qiagenbioinformatics.com/products/ingenuity-pathway-analysis)^32^. The results from the challenged vs. nonchallenged Leghorns at 6 dpi contrast were input into IPA, transcripts with FDR <0.05 and absolute LFC >1 were used for an expression analysis. Figure 3 was generated based on the upstream regulator TCR (activation z-score = 2.712; p-value = 7.52E-10) from the upstream analysis portion in IPA. Additional diseases & functions (p-value <0.0001) relevant to NDV challenge were added to the network using the grow function (Fig. 3). The results from the Fayoumi vs. Leghorn contrasts were input into IPA and transcripts with FDR <0.10 were used for a comparison analysis for both challenged and nonchallenged birds at 2 and 6 dpi (Fig. 4); no pathways were significantly different between the two lines at 10 dpi. IPA calculates a z-score based on the expression levels of genes within a pathway or related to a functional term. If a z-score is positive IPA predicts activation of a pathway or function, and if negative IPA predicts inhibition of a pathway or function.

### Method validation

To validate the RNA-seq methodology, high-throughput qPCR using the Fluidigm Biomark system was performed. The methods^17,18^ and primers^17^ were reported previously. The LFC calculated by edgeR for the RNA-seq data and the −2^−ΔΔCT^ method from the Fluidigm Biomark output was used for the correlation of the challenged vs. nonchallenged comparisons at each time and within each line for 32 genes, resulting in 192 data points.

## Electronic supplementary material

Supplementary Tables S1-3

## References

[1] Wight PAL, Burns RB, Rothwell B, Mackenzie GM (1971). The Harderian gland of the domestic fowl I. Histology, with reference to the genesis of plasma cells and Russell bodies. J. Anat..

[2] Bang BG, Bang FB (1968). Localized lymphoid tissues and plasma cells in paraocular and paranasal organ systems in chickens. Am J Pathol.

[3] Mueller AP, Sato K, Glick B (1971). The chicken lacrimal gland, gland of Harder, caecal tonsil, and accessory spleens as sources of antibody-producing cells. Cellular Immunology.

[4] Burns RB, Maxwell MH (1978). The structure of the Harderian and lacrimal gland ducts of the turkey, fowl and duck. A light microscope study. J. Anat..

[5] Burns RB (1976). Specific antibody production against a soluble antigen in the Harderian gland of the domestic fowl. Clin. exp. Immunol..

[6] Manisikka A (1989). B cell maturation in the chicken Harderian gland. The Journal of Immunology.

[7] Davelaar FG, Kouwenhoven B (1976). Changes in the Harderian gland of the chicken following conjunctival and intranasal infection with infectious bronchitis virus in one- and 20-day-old chickens. Avian Pathol.

[8] Survashe BD, Aitken ID, Powell JR (1979). The response of the Harderian gland of the fowl to antigen given by the ocular route. I. Histological changes. Avian Pathol.

[9] Sundick RS, Albini B, Wick G (1973). Chicken Harder’s gland: evidence for relatively pure bursa-dependent lymphoid cell population. Cellular Immunology.

[10] Gallego M, Glick B (1988). The proliferative capacity of the cells of the avian Harderian gland. Dev Comp Immunol.

[11] Maslak, D. M. *Head-associalted lymphoid tissue [HALT] of the chicken: characterization of lymphocytes Doctor of Philosophy thesis*, Iowa State University (1994).

[12] Russell PH, Dwivedi PN, Davison TF (1997). The effects of cyclosporin A and cyclophosphamide on the populations of B and T cells and virus in the Harderian gland of chickens vaccinated with the Hitchner B1 strain of Newcastle disease virus. Veterinary Immunology and Immunopathology.

[13] Kitalyi AJ (1998). Village chicken production systems in rural Africa household food security and gender issues. FAO Animal Production and Health Paper.

[14] Hassan MK, Afify MA, Aly MM (2004). Genetic resistance of Egyptian chickens to infectious bursal disease and Newcastle disease. Tropical Animal Health and Production.

[15] Cole RK, Hutt FB (1961). Genetic differences in resistance to Newcastle disease. Avian Diseases.

[16] Albiston HE, Gorrie CJR (1942). Newcastle disease in Victoria. Aust. Vet. J..

[17] Deist MS (2017). Novel mechanisms revealed in the trachea transcriptome of resistant and susceptible chicken lines following infection with Newcastle disease virus. Clin Vaccine Immunol.

[18] Deist MS (2017). Resistant and susceptible chicken lines show distinctive responses to Newcastle disease virus infection in the lung transcriptome. BMC Genomics.

[19] Zhang J (2018). Transcriptome Analysis in Spleen Reveals Differential Regulation of Response to Newcastle Disease Virus in Two Chicken Lines. Sci Rep.

[20] Liu H, Kung H, Fulton JE, Morgan RW, Cheng HH (2001). Growth hormone interacts with Marek’s disease virus SORF2 protein and is associated with disease ressitance in chicken. Pnas.

[21] Ewert DL, Barger BO, Eidson CS (1979). Local antibody response in chickens: analysis of antibody synthesis to Newcastle disease virus by solid-phase radioimmunoassay and immunofluorescence with class-specific antibody for chicken immunoglobulins. Infection and Immunity.

[22] Nagai Y (1995). Virus activation by host proteinases. A pivotal role in the spread of infection, tissue tropism and pathogenicity. Microbiol. Immunol..

[23] Campos, R. K. *et al*. RPLP1 and RPLP2 Are Essential Flavivirus Host Factors That Promote Early Viral Protein Accumulation. *J Virol* **91**, 10.1128/JVI.01706-16 (2017). 10.1128/JVI.01706-16 PMC5286887 27974556

[24] Ng TH, Chiang YA, Yeh YC, Wang HC (2014). Review of Dscam-mediated immunity in shrimp and other arthropods. Dev Comp Immunol.

[25] Fialka F (2008). CPA6, FMO2, LGI1, SIAT1 and TNC are differentially expressed in early- and late-stage oral squamous cell carcinoma–a pilot study. Oral Oncol.

[26] Kim D (2013). TopHat2: accurate alignment of transcriptomes in the presence of insertions, deletions and gene fusions. Genome Biol.

[27] Anders S, Pyl PT, Huber W (2015). HTSeq–a Python framework to work with high-throughput sequencing data. Bioinformatics.

[28] Li H, Durbin R (2010). Fast and accurate long-read alignment with Burrows-Wheeler transform. Bioinformatics.

[29] Marini, F. pcaExplorer: interactive visualization of RNA-seq data using a principal components approach (2016).

[30] Love MI, Huber W, Anders S (2014). Moderated estimation of fold change and dispersion for RNA-seq data with DESeq. 2. Genome Biol.

[31] Robinson MD, McCarthy DJ, Smyth G (2010). K. edgeR: a Bioconductor package for differential expression analysis of digital gene expression data. Bioinformatics.

[32] Kramer A, Green J, Pollard J, Tugendreich S (2014). Causal analysis approaches in Ingenuity Pathway Analysis. Bioinformatics.

